# Targeting the miR-6734-3p/ZEB2 axis hampers development of non-small cell lung cancer (NSCLC) and increases susceptibility of cancer cells to cisplatin treatment

**DOI:** 10.1080/21655979.2021.1936891

**Published:** 2021-06-09

**Authors:** Linying Wei, Jianyang Jiang

**Affiliations:** a Department of Respiratory and Critical Care Medicine, People’s Hospital of Changshan, Quzhou City, Zhejiang Province, China; b Department of Respiratory and Critical Care Medicine, People’s Hospital of Quzhou, Quzhou City, Zhejiang Province, China

**Keywords:** Non-small cell lung cancer, miR-6734-3p, zinc finger E-box binding homeobox 2, cisplatin, chemosensitivity

## Abstract

The unclear pathogenesis mechanisms and resistance of cancer cells to chemical drugs serious limits the development of effective treatment strategies for non-small cell lung cancer (NSCLC). In this study, we managed to investigate this issue, and identify potential cancer associated biomarkers for NSCLC diagnosis, prognosis and treatment. This study found that miR-6734-3p was downregulated in both NSCLC clinical specimens (tissues and serum) and cells, compared to the normal tissues and cells. Next, upregulation of miR-6734-3p inhibited cancer formation and progression in NSCLC cells *in vitro* and *in vivo*. Conversely, miR-6734-3p ablation had opposite effects and facilitated NSCLC development. In addition, miR-6734-3p bound to the 3ʹ untranslated region (3ʹUTR) of zinc finger E-box binding homeobox 2 (ZEB2) mRNA to suppress its expressions in NSCLC cells. Interestingly, the inhibiting effects of miR-6734-3p overexpression on NSCLC progression were abrogated by upregulating ZEB2. Furthermore, both upregulated miR-6734-3p and silencing of ZEB2 increased cisplatin-sensitivity in cisplatin-resistant NSCLC (CR-NSCLC) cells. Taken together, miR-6734-3p played an anti-tumor role to hinder cancer development and enhanced the cytotoxic effects of cisplatin treatment on NSCLC cells by downregulating ZEB2.

## Graphical Abstract

**Figure:**
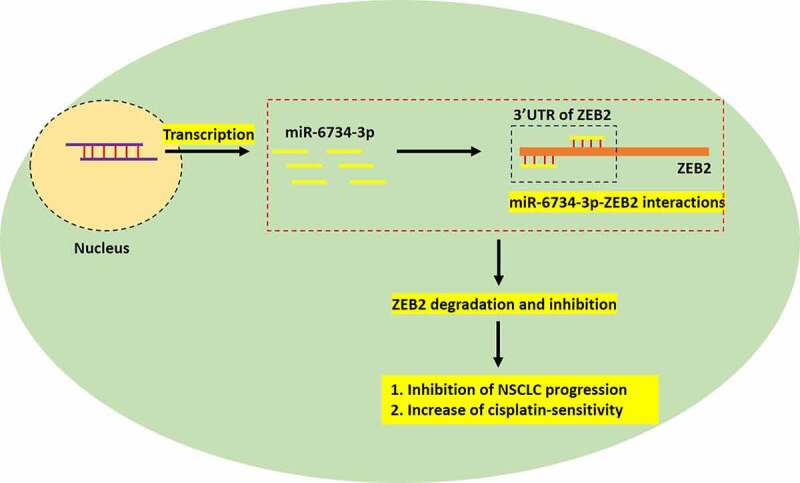

## Introduction

As a common aggressive malignancy among human beings, non-small cell lung cancer (NSCLC)-related deaths seriously degrades the life quality of human beings worldwide, especially in China [1–3]. The molecular mechanisms of NSCLC pathogenesis are very complicated, which is the results of the interactions among multiple cancer associated genes, including oncogenes and tumor suppressors [4–6]. Currently, our knowledge in NSCLC progression is very poor, and identification of its novel molecular mechanisms may help us to improve NSCLC patients’ outcome [7–9]. Dysregulated microRNAs (miRNAs) are recently identified as biomarkers for cancer progression [10,11], and targeting cancer associated miRNAs are proved to be effective strategies to slow down NSCLC progression [12–14]. Among all the miRNAs, this study selected miR-6734-3p for further investigations, and the precursor miR-6734-3p locates in chr1:43,364,648–43,364,715 (-). According to the previous publications [15,16], miR-6734-3p acts as a tumor suppressor to inhibit cancer progression in colon cancer as well as acute myeloid leukemia; however, the regulating mechanisms of miR-6734-3p in other types of cancer, including NSCLC, is still largely unknown.

Based on recent publications [17,18], miRNAs exert their biological functions through binding to the 3ʹ untranslated region (3ʹUTR) of the downstream genes mRNA, resulting in mRNA instability and degradation [19,20]. Our preliminary analysis suggested that zinc finger E-box binding homeobox 2 (ZEB2) was the downstream target of miR-6734-3p, which renders the possibility that the miR-6734-3p/ZEB2 axis may participate in regulating NSCLC pathogenesis. Based on the information from public database, we assure that ZEB2 exerts its tumor-promoting effects in NSCLC [21–23]. For example, Jiangying Cui et al. showed that ZEB2 activates Wnt/β-catenin pathway to facilitate NSCLC progression ^[23]^, Yan Wang et al. proved that ZEB2 promotes epithelial-mesenchymal transition (EMT) ^[22]^ and Xiangdong Tong et al. evidenced that ZEB2 promotes cell proliferation and growth ^[21]^ in NSCLC. In addition, ZEB2 can be negatively regulated by multiple miRNAs in NSCLC [21,24,25]; however, no publications report the regulatory mechanisms between miR-6734-3p and ZEB2, which makes this issue become novel and meaningful.

Furthermore, although the great advances have been reached in NSCLC treatment strategies, the factors, for example, drug resistance and tumor metastasis, have limited their therapeutic efficacy in clinical practices [26–29]. Among all the drugs, cisplatin (DDP) is the first-line chemical drug for NSCLC treatment in clinic [30,31], and the development of novel strategies to eliminate cisplatin-resistance in NSCLC become urgent and necessary. Interestingly, recent data suggested that targeting cancer-associated miRNAs is capable of improving the cisplatin-sensitivity in NSCLC, these miRNAs include miR-219a-5p ^[32]^, miR-140-5p ^[33]^, miR-608 ^[34]^, et al. Nevertheless, it is still unclear whether targeting miR-6734-3p alters cisplatin-resistance in NSCLC. Interestingly, ZEB2 involves in regulating cisplatin-chemosensitivity in NSCLC, and silencing of ZEB2 increases cisplatin-sensitivity in NSCLC cells [32,33].

In general, we purposed that the miR-6734-3p/ZEB2 axis might play an important role in regulating NSCLC progression and drug resistance, hence, the present study was designed to investigate the regulating effects and underlying mechanisms of this signal pathway in regulating NSCLC development and cisplatin-resistance, which will provide novel biomarkers for NSCLC diagnosis and treatment.

## Materials and methods

### Collection of the clinical samples

The NSCLC patients (N = 50) were recruited in People’s Hospital of Changshan from 2014 to 2017, and the cancerous and adjacent non-cancerous tissues were obtained by surgery, and the serum was collected from NSCLC patients and normal volunteers, all the above clinical specimens were immediately stored at −70°C refrigerator, all the tissues were judged as eligible by two experienced pathologists in our hospital. Before surgical resection, we assured that all the patients were not subjected to other types of therapies. The tissues were used for further real-time qPCR and Western Blot analysis. All the patients had signed the informed consent forms, and the ethics approval had been granted by the People’s Hospital of Changshan Ethics Committee.

### Cultivation and treatments for cells

We purchased the NSCLC cells (A549, H1299, SK-MES-1 and Calu-3) and normal HBE cells from American Type Culture Collection (ATCC, USA) and the cells were cultivated in Roswell Park Memorial Institute 1640 medium (RPMI-1640, Gibco, USA) containing 10% fetal bovine serum (FBS, Gibco, USA). All the above cells were maintained in the conditions with 5% CO_2_ humidified air at 37°C. Next, the NSCLC cells were exposed to continuous low-dose cisplatin treatments (0.5–5 μg/ml) for 80 days to induct cisplatin-resistant descent NSCLC cells (CR-NSCLC). Finally, the CR-NSCLC cells were challenged by high-dose cisplatin with 25 μg/ml for differential time points (0 h, 24 h, 48 h and 72 h).

### Vector transfection

The commercial third-party companies, including Sangon Biotech (Shanghai, China) and Ribio (Guangzhou, China) were entrusted to design and construct the mimic and inhibitor for miR-6734-3p, and the gene manipulating vectors for wild-type and mutant ZEB2, respectively. The sequences of miR-6734-3p mimic and inhibitor could be found in the previous studies [15,16]. After that, the above mimic, inhibitor and plasmids were delivered into the NSCLC cells by using the Lipofectamine 2000 reagent (Invitrogen, USA) following the producer’s instruction. At 48 h post-transfection, real-time qPCR was conducted to validate the transfection efficiency of the above vectors.

### Real-Time qPCR

Total RNA was extracted by using a Trizol kit purchased from Invitrogen (USA), and the Real-Time qPCR kit (QIAGEN, Germany) was employed to examine relative gene expressions, and miR-6734-3p was normalized to U6, while ZEB2 mRNA was normalized to β-actin. The primer sequences could be found from the previous publications [15,16,21–23].

### Western Blot analysis

The NSCLC cells with or without genes manipulation and clinical tissues were lysed and the total proteins were extracted by using the RIPA lysis buffer bought from Beyotime (Shanghai, China) in keeping with the producer’s instruction, and the subsequent SDS-PAGE was conducted to separate proteins according their molecular weight. Next, the proteins were transferred onto the PVDF membranes (Millipore, USA), which were blocked by 5% slim milk for 40 min at room temperature. Finally, after primary and secondary antibodies incubation, the enhanced chemiluminescent (ECL) reagent (Sigma, USA) was used for protein visualization, which were quantified by using the Image J software. The information for the primary antibodies against β-actin (1:1500, #ab8226, Abcam, USA), ZEB2 (1:2000, #ab138222, Abcam, USA), N-cadherin (1:1500, #ab98952, Abcam, USA) and Vimentin (1:1500, #ab8069, Abcam, USA).

### Examination of cell proliferation, colonies formation abilities and viability

Cell proliferation and colonies formation abilities were evaluated by using the CCK-8 assay and colony formation assay, respectively. For cell proliferation, the CCK-8 reagent (AbMole, USA) was purchased, and the NSCLC cells were maintained in the 96-well plates for 0 h, 24 h, 48 h and 72 h, and the cells were incubated with CCK-8 working reagent in the refrigerator with standard culture conditions for 4 h. Next, the plates were vortexed and cell proliferation abilities were measured by a Gemini EM microplate reader (ThermoFisher Scientific, USA). For colonies formation abilities, the NSCLC cells were cultured in 6-well plates for 14 days at the density of 1000 cells/well, and the colonies were stained with 0.1% crystal violet, observed and counted under a light microscope (ThermoFisher Scientific, USA). For cell viability, the NSCLC cells were stained with trypan blue staining solution for 5 min at 37°C, and the dead blue cells were recorded under a light microscope.

### Examination of cell apoptosis by flow cytometry (FCM) assay

Here we purchased a commercial Apoptosis Detection kit (BD Bioscience, USA) to examine the apoptosis ratio of NSCLC cells in keeping with the experimental procedures provided by the manufacturer. Briefly, the NSCLC cells were exposed to differential treatments, washed by PBS, and double-stained with the Annexin V-FITC and PI dye for 25 min at room temperature in darkness. Then, the cells stained by the above two dyes were monitored and quantified by using a flow cytometer (ThermoFisher Scientific, USA).

### Examination of cell invasion and migration

Here we conducted a transwell assay and wound scratch assay to examine cell invasion and migration abilities, respectively. For the transwell assay, the NSCLC cells were cultured with FBS-free medium in the upper chamber of the transwell plates (BD Biosciences, USA), and the lower chambers were full of RPMI-1640 medium containing 10% FBS (Gibco, USA) as chemoattract. At 24 h post-culture, the cells in the upper surface of the Matrigel were removed, and cells in the lower chamber were stained by 0.1% crystal violet and the cells were photographed and counted under light microscope to evaluate cell invasion. For the wound scratch assay, the NSCLC cells were cultured in the six-well plates (5 × 10^5^ cells/well) for 24 h when the cell confluency reached about 95%, and a 100 μl tip was used to generate wound scratches with equal distance at 0 h. After 24 h post-culture, and distances of the wound scratches were calculated under a light microscope.

### Verification of the binding sites in miR-6734-3p and 3ʹ UTR of ZEB2 mRNA

Here we, respectively, used dual-luciferase reporter gene system assay and RNA pull-down assay to verify the targeting sites in miR-6734-3p and ZEB2 mRNA. We initially performed dual-luciferase reporter gene system assay according to previous work [15,16]. Specifically, the targeting sites were predicted by the online miRDB software (http://mirdb.org/), and the binding sites in ZEB2 mRNA were mutated and cloned into the luciferase reporter plasmids by Sangon Biotech (Shanghai, China). The mutant or wild-type plasmids were co-cultured with miR-6734-3p mimic and inhibitor into the NSCLC cells, and the relative luciferase activities were measured by using the Luciferase Assay kit (Promega, USA).

### RNA pull-down assay

The RNA pull-down assay was conducted. Briefly, the Sangon Biotech (Shanghai, China) was entrusted to construct the ZEB2 mRNA labeled with biotin, which were used to enrich miR-6734-3p in the lysates of the NSCLC cells by using the ZEB2 probe-streptavidin Dynabeads (Invitrogen, USA). Then, the enrichment of miR-6734-3p was evaluated by using the following Real-Time qPCR analysis.

### Xenograft tumor-bearing mice models

The BALB/c nude mice (Aged 6–8 weeks, male) were purchased from the Research Animal Center of Zhejiang University, and all the mice were fed under specific pathogen-free (SPF) conditions with freely accessible to food and water, and with 12 h dark-and-light circle. Then, the NSCLC cells were diluted by PBS, and were subcutaneously injected into the dorsal flank of the mice, and were at the concentration of 5 × 10^6^ cells per mouse. Next, the tumor volumes were monitored every 5 days from day 0 to day 25. Finally, at day 25 after the tumor volumes were measured, the mice were anesthetized and sacrificed to obtain the mice tumors, which were weighed and the tumor tissues were prepared for further analysis. We had got the approval for animal experiments from the Ethics Committee of People’s Hospital of Changshan.

### *Immunohistochemistry (IHC) analysis for Ki67 protein* in vivo

We obtained the mice tumor tissues, which were fixed by using the 10% (v/v) formaldehyde and the tissues were subsequently embedded into the paraffin. Then, the above tissues were prepared as sections with about 5 μm thickness. After that, according to the protocols provided by the previous publication [34], we performed IHC assay to examine the expression status, including expression levels and localization of Ki67 protein in mice tumor tissues. Briefly, the tissues were sequentially incubated with primary antibody against Ki67 (1:400, #ab245113, Abcam, UK) and secondary antibody (Cell signaling Technology, USA), and the yellow Ki67-postive cells were observed and counted under a light microscope (ThermoFisher Scientific, USA).

### Data collection, analysis and visualization

The original data were collected, processed and indicated as Means ± Standard Deviation (SD) format. Here we used the SPSS 18.0 software for data analysis and GraphPad Prism 8.0 software for data presentation. Comparisons between two means were conducted by the Student’s t-test, and means in multiple groups were compared by using the one-way ANOVA analysis. In addition, patient’s prognosis was predicted by the Kaplan-Meier survival analysis, and genes correlations were analyzed by Pearson Correlation Analysis in the clinical samples. Individual experiment had 3 repetitions, and *P < 0.05.

## Results

### Correlations and expressions of miR-6734-3p and ZEB2 in NSCLC clinical tissues and cell lines

**Figure 1.**
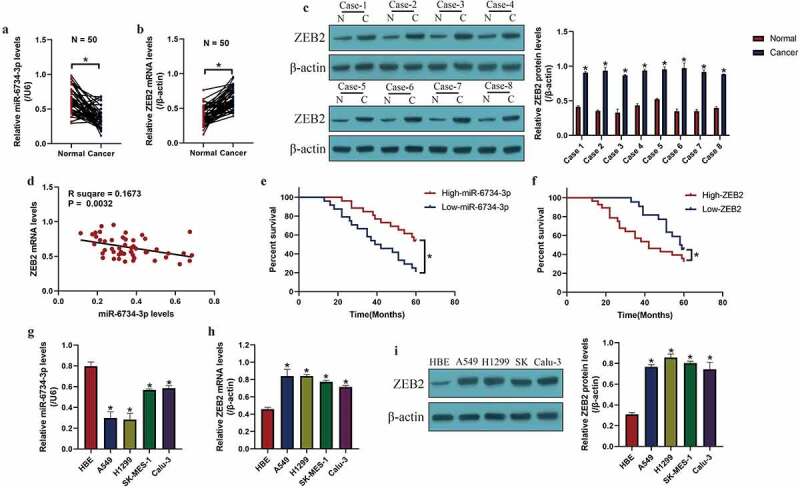
MiR-6734-3p and ZEB2 were aberrantly expressed in NSCLC clinical specimens and cells. The levels of (a) miR-6734-3p and (b) ZEB2 mRNA in NSCLC tissues were examined by real-time qPCR. (c) Eight patients were randomly selected, and ZEB2 protein levels in NSCLC tissues were measured by Western Blot. (d) The correlations between miR-6734-3p and ZEB2 mRNA in NSCLC tissues were analyzed by using the pearson correlation analysis. The correlations of (e) miR-6734-3p and (f) ZEB2 mRNA with patient prognosis were analyzed. The expressions of (g) miR-6734-3p and (h) ZEB2 mRNA were determined in NSCLC cells. (i) ZEB2 expressions at protein levels in NSCLC cells were examined. Individual experiment had 3 repetitions, and *P < 0.05

According to recent data, miR-6734-3p hindered cancer progression in colon cancer ^[16]^ and acute myeloid leukemia ^[15]^, but the tumor-regulating effects of miR-6734-3p in other types of cancer were still not investigated. Hence, this study focused on investigating this issue in NSCLC. Initially, the clinical tissues (N = 50), including cancerous and non-cancerous tissues were obtained, and the expressions of miR-6734-3p and ZEB2 were measured by using Real-Time qPCR (Figure 1(a,b) and Western Blot analysis (Figure 1(c)). The results in Figure 1(a-c) indicated that miR-6734-3p was downregulated (*P* < 0.05), while ZEB2 was upregulated (*P* < 0.05) in cancer tissues but not in their paired normal tissues. Consistently, data in Figure S3 supported that miR-6734-3p was also downregulated in NSCLC patients’ serum (N = 20), in contrast with the normal volunteers (N = 20). In addition, as indicated in Figure 1(d), miR-6734-3p was negatively relevant to ZEB2 mRNA in NSCLC tissues (*P* = 0.0032). Next, the follow-up visit experiments were conducted for 60 months, and the analysis results indicated that NSCLC patients with low-expressed miR-6734-3p (*P* < 0.05, Figure 1(e)) and high-expressed ZEB2 (*P* < 0.05, Figure 1(f)) tended to have an unfavorable prognosis. Consistently, the cellular experiments were performed, and we found that NSCLC cells (A549, H1299, SK-MES-1 and Calu-3) were featured by lower levels of miR-6734-3p (*P* < 0.05, Figure 1g) and higher levels of ZEB2 (*P* < 0.05, Figure 1(h,i)), in contrast with the normal HBE cells, which were in line with our clinical results. In addition, since miR-6734-3p was prone to be low-expressed in A549 and H1299 cells (Figure 1(g)), we chosen the two cells for further analysis.

### *MiR-6734-3p exerted its tumor-inhibiting effects in NSCLC* in vitro *and* in vivo

**Figure 2.**
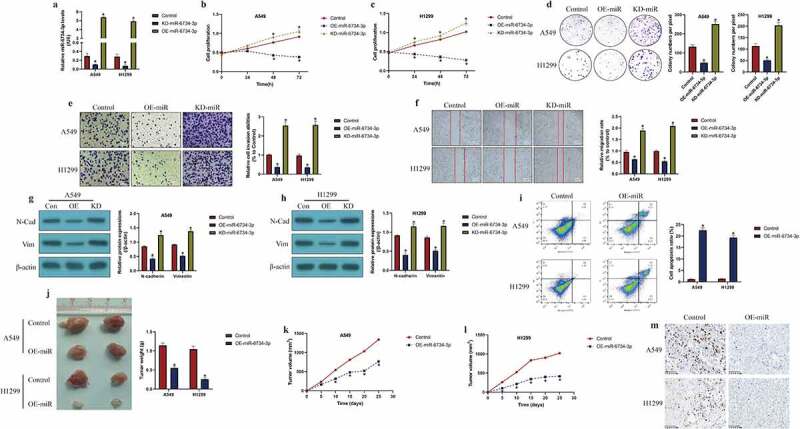
The effects of miR-6734-3 on the malignant phenotypes in NSCLC cells. (a) Delivery of miR-6734-3p mimic and inhibitor into the NSCLC cells. (b, c) At 0 h, 24 h, 48 h and 72 h post-culture, CCK-8 assay was conducted to evaluate cell proliferation. (d) Colony formation assay was performed to reflect cell growth. (e, f) The transwell assay and wound scratch assay were performed to evaluated cell mobility. (g, h) The protein levels of EMT associated biomarkers were examined by Western Blot. (i) Measurement of cell apoptosis ratio by FCM. *In vivo* animal experiments validated that miR-6734-4p inhibited (j) tumor weight and (k, l) volume in animal models. (m) Localization and expressions of Ki67 protein were examined by IHC in mice tumor tissues. Individual experiment had 3 repetitions, and *P < 0.05

Further experiments were conducted to determine whether miR-6734-3p was associated with NSCLC pathogenesis. To conduct further gain- and loss-of-function experiments, the miR-6734-3p mimic and inhibitor were constructed and delivered into A549 and H1299 cells for its overexpression and downregulation (*P* < 0.05, Figure 2(a)), respectively. The results in Figure 2(b-d) evidenced that overexpression of miR-6734-3p suppressed cell proliferation (*P* < 0.05, Figure 2(b,c)) and colony formation abilities (*P* < 0.05, Figure 2(d)) in a time-dependent manner, while miR-6734-3p ablation had opposite effects (*P* < 0.05, Figure 2(b-d)). Also, we identified that miR-6734-3p influenced cell mobility in a similar manner, and the evidences supported that upregulation of miR-6734-3p inhibited cell invasion (*P* < 0.05, Figure 2(e)) and migration (*P* < 0.05, Figure 2(f)), which were promoted by downregulating miR-6734-3p, as indicated in Figure 2(e) by transwell assay and Figure 2(f) by wound scratch assay, respectively. Consistently, our results also indicated that miR-6734-3p downregulated N-cadherin and Vimentin to suppress epithelial-mesenchymal transition (EMT) in NSCLC cells (*P* < 0.05, Figure 2(g,h)). Next, the FCM results indicated that miR-6734-3p overexpression increased cell apoptosis ratio in NSCLC (*P* < 0.05, Figure 2(i)). Finally, we used NSCLC cells to establish tumor-bearing mice models *in vivo*, and the following experiments validated that miR-6734-3p slowed down tumor growth in mice (*P* < 0.05, Figure 2(j-l)) and downregulated Ki67 expression levels (Figure 2(m)) in mice tumor tissues *in vivo*.

### MiR-6734-3p negatively regulated ZEB2 expressions in NSCLC cells

**Figure 3.**
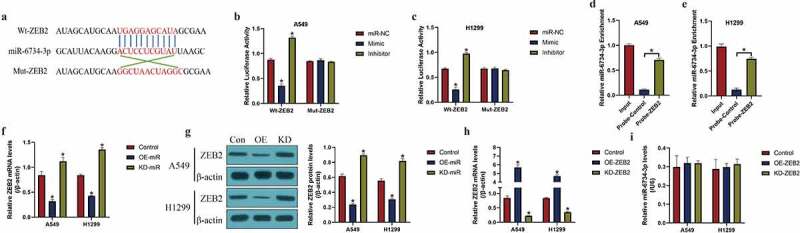
The regulatory mechanisms of miR-6734-3p and ZEB2. (a) The targeting sites in miR-6734-3p and ZEB2 mRNA were predicted. (b, c) dual-luciferase reporter gene system assay and (d, e) RNA pull-down assay was utilized to validate the binding sites of miR-6734-3p and ZEB2. The (f) mRNA and (g) protein levels of ZEB2 could be negatively regulated by miR-6734-3p. (h) The overexpression and silencing vectors for ZEB2 were delivered into NSCLC cells, and (i) manipulation of ZEB2 had little effects on miR-6734-3p expressions in NSCLC cells. Individual experiment had 3 repetitions, and *P < 0.05

Next, we investigated the regulatory mechanisms of miR-6734-3p and ZEB2 in NSCLC cells. To achieve this, the online miRDB software (http://mirdb.org/) was used to predict the targeting sites of miR-6734-3p and 3ʹ untranslated region (UTR) of ZEB2 mRNA (Figure 3(a)). By conducting the following dual-luciferase reporter gene system assay, we validated the above predicted binding sites in A549 and H1299 cells (Figure 3(b,c)). Specifically, wild-type (Wt) and mutant (Mut) ZEB2 sequences were cloned into luciferase reporter plasmids for vectors construction, and were co-transfected with the miR-6734-3p mimic and inhibitor into the NSCLC cells. We expectedly found that the relative luciferase activities in NSCLC co-transfected with Wt-ZEB2, instead of Mut-ZEB2, were negatively influenced by miR-6734-4p (*P* < 0.05, Figure 3(b,c)). Similarly, further RNA pull-down assay results supported that miR-6734-3p was significantly enriched by ZEB2 probes labeled with biotin (*P* < 0.05, Figure 3(d,e)). Furthermore, by conducting the Real-Time qPCR (Figure 3(f)) and Western Blot analysis (Figure 3(g)), we validated that miR-6734-3p suppressed ZEB2 expressions in NSCLC cells (*P* < 0.05), while manipulation of ZEB2 had little effects on miR-6734-3p expressions (*P* > 0.05, Figure 3(h,i)).

### Overexpression of miR-6734-3p degraded ZEB2 restrain NSCLC development

**Figure 4.**
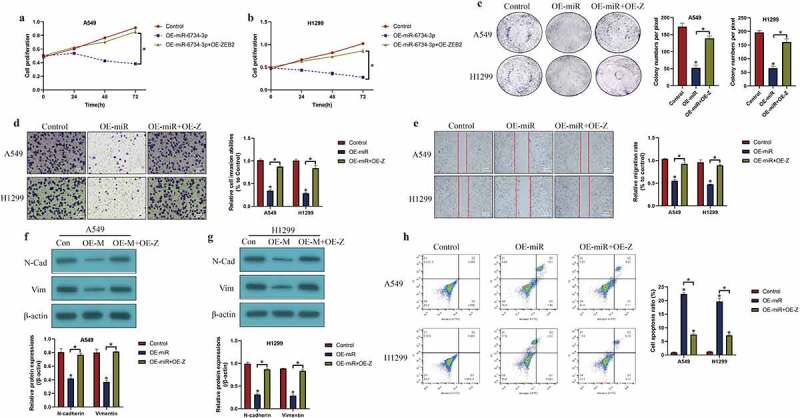
Targeting the miR-6734-3p/ZEB2 axis hampered cancer progression in NSCLC. (a, b) CCK-8 assay and (c) colony formation assay was used to evaluated cell growth *in vitro*. Cell mobility was evaluated by using the (d) Transwell assay and (e) wound scratch assay. (f, g) The protein levels of EMT associated signatures were determined by Western Blot. (h) Cell apoptosis ratio in NSCLC cells was measured by FCM assay. Individual experiment had 3 repetitions, and *P < 0.05

Given that ZEB2 and miR-6734-3p played opposite regulating role in NSCLC progression [23,35], and there existed regulating relationship between these two genes, we explored whether miR-6374-3p regulated NSCLC development through targeting ZEB2. To validate this hypothesis, we determined the effects of miR-6734-3p/ZEB2 axis on the malignant phenotypes in NSCLC. Specifically, as indicated in Figure 4(a-c), miR-6734-3p overexpression suppressed cell proliferation (*P* < 0.05, Figure 4(a), b) and colonies formation abilities (*P* < 0.05, Figure 4(c)), which were restored by upregulating wild-type ZEB2 (*P* < 0.05, Figure 4(a-c)). However, the MTT assay results in Figure S2A-B suggested that upregulation of mutant ZEB2 did not influence the effects of miR-6734-3p overexpression on cell proliferation. In addition, miR-6734-3p also regulated cell mobility by targeting ZEB2 (Figure 4(d,e)). As shown in Figure 4(d,e), the transwell assay and wound scratch assay results suggested that the inhibiting effects of miR-6734-3p upregulation on cell invasion and migration were abrogated by overexpressing ZEB2 (*P* < 0.05, Figure 4(d,e)). Similarly, ZEB2 overexpression abrogated the inhibiting effects of miR-6734-3p on the expression levels of the EMT associated biomarkers (N-cadherin and Vimentin) (*P* < 0.05, Figure 4(f,g)). Finally, the FCM results provided evidences to support that upregulation of miR-6734-3p silenced ZEB2 to trigger cell apoptosis in NSCLC cells (*P* < 0.05, Figure 4(h)).

### Targeting the miR-6734-3p/ZEB2 axis changed cisplatin-resistance in NSCLC cells

**Figure 5.**
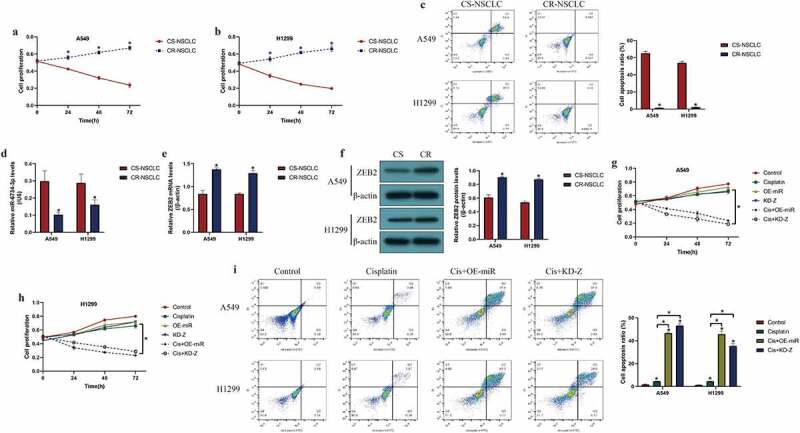
Overexpression of miR-6734-3p sensitized NSCLC cells to cisplatin treatment. The CS-NSCLC and CR-NSCLC cells were challenged by high-dose cisplatin. (a, b) Cell proliferation was determined by CCK-8 assay. (c) FCM was performed to examine cell apoptosis. (d) Downregulated miR-6734-3p, and (e, f) upregulated ZEB2 were observed in CR-NSCLC cells, in contrast with the corresponding CS-NSCLC cells. (g, h) Cisplatin-induced inhibition of NSCLC cell proliferation was enhanced by miR-6734-3p overexpression and ZEB2 ablation. (i) Targeting miR-6734-3p and ZEB2 increased cell apoptosis ratio in CR-NSCLC cells stimulated by high-dose cisplatin. Individual experiment had 3 repetitions, and *P < 0.05

Cisplatin had been commonly used for NSCLC treatment in clinic [31,36], and dysregulated miRNAs were closely associated with drug resistance of cisplatin in NSCLC [37,38]; hence, further experiments were designed to investigate the role of the miR-6734-3p/ZEB2 pathway in regulating cisplatin-resistance in NSCLC. The cisplatin-resistant NSCLC (CR-NSCLC) cells (A549/DDP, H1299/DDP) were inducted by exposing the parental cisplatin-sensitive NSCLC (CS-NSCLC) cells (A549 and H1299) to continuous cisplatin treatment, and the above cells were subsequently challenged by high-dose cisplatin. As expected, the results in Figure 5(a-c) suggested that CR-NSCLC cells but not CS-NSCLC cells were more insensitive to high-dose cisplatin stimulation (*P* < 0.05, Figure 5(a-c)). Next, our data indicated that miR-6734-3p and ZEB2 were also influenced by cisplatin treatment, and the results showed that continuous low-dose cisplatin pressure suppressed miR-6734-3p (*P* < 0.05, Figure 5(d)), while elevated ZEB2 in CR-NSCLC cells (*P* < 0.05, Figure 5(e)). Finally, as shown in Figure 5(g-i), we proved that both miR-6734-3p overexpression and ZEB2 ablation restrained cell proliferation (*P* < 0.05, Figure 5(g,h)) and induce cell apoptosis (*P* < 0.05, Figure 5(i)) to sensitize CR-NSCLC cells to cisplatin stimulation, which were examined by CCK-8 assay and FCM assay, respectively. In addition, the results in Figure S1A-B supported that overexpression of ZEB2 increased cisplatin-resistance in CS-NSCLC cells (*P* < 0.05).

## Discussion

Recent data suggested that dysregulated miRNAs are closely associated with the non-small cell lung cancer (NSCLC) pathogenesis [12–14], and targeting cancer-related miRNAs is effective to slow down cancer progression [12–14]. According to recent publications, miR-6734-3p exerts its tumor-suppressing effects in colon cancer ^[16]^ and acute myeloid leukemia ^[15]^, but its role in regulating the pathogenesis of other types of cancer, such as NSCLC, has not been reported. Analysis of the data in the present work suggested that miR-6734-3p was aberrantly deficient in the NSCLC tissues, serum and cells, and high-expressed miR-6734-3p predicted a favorable prognosis in patients with NSCLC, indicating that miR-6734-3p was closely associated with NSCLC progression, which were supported by the previous work in other types of cancers [15,16]. Also, we noticed that miR-6734-3p functioned as a tumor suppressor to reverse the aggressiveness of NSCLC cells *in vitro* and *in vivo*, implying that targeting miR-6734-3p was effective to slow down NSCLC progression, which were in line with the previous work [15,16], and evidenced that miR-6734-3p could serve as a diagnostic and prognostic biomarker for NSCLC.

Given that miRNAs exert their biological functions through targeting the 3ʹ UTR of the downstream target genes [17,18], and our data screened out that miR-6734-3p served as a potential upstream regulator for ZEB2. According to the biological functions of miRNAs [17,18], we first verified that miR-6734-3p targeted the 3ʹ UTR of ZEB2 mRNA for its inhibition and degradation in NSCLC cells. In addition, ZEB2 acted as an oncogene to facilitate the progression of NSCLC [21–23], and our data supported that ZEB2 was aberrantly overexpressed in NSCLC tissues and cells, and high-expressed ZEB2 predicted a worse prognosis in NSCLC patients. In addition, our data suggested that ZEB2 mRNA and miR-6734-3p were negatively correlated in the NSCLC tissues. Based on the fact that there existed regulatory mechanisms between miR-6734-3p and ZEB2, and they had opposite effects in regulating cancer progression [15,16,21–23], our data evidenced that miR-6734-3p targeted ZEB2 to hinder the development of NSCLC, implying that targeting the miR-6734-3p/ZEB2 axis was a novel strategy to treat NSCLC.

Drug resistance gradually become an urgent issue that seriously blocks the utilization and effectiveness of chemical drugs for NSCLC treatment in clinic, and up until now, those problems have not been solved, which cause high-mortality in NSCLC patients [26–28]. Among all the chemical drugs, cisplatin is most commonly used for NSCLC treatment in clinic [30,31]. Recent publications suggested that long-term cisplatin pressure-induced miRNA-mRNA networks dysregulation occurs during the generation of cisplatin-resistance in NSCLC [39–41], and this study validated that manipulation of miR-6734-3p/ZEB2 axis improved cisplatin-sensitivity in NSCLC cells *in vitro*. Functionally, continuous low-dose cisplatin inhibited miR-6734-3p, while promoted ZEB2 expressions in CR-NSCLC cells, suggesting that continuous low-dose cisplatin exposure altered miR-6734-3p and ZEB2 expression patterns in CR-NSCLC cells. In addition, we found that both miR-6734-3p overexpression and ZEB2 downregulation increased the susceptibility of NSCLC cells to cisplatin stimulation, which were partly supported by the previous data [32,33] and suggested that miR-6734-3p and ZEB2 could be used as potential agents to improve cisplatin-sensitivity for NSCLC treatment in clinic. This study provided enough data to support that the miR-6734-3p/ZEB2 axis was crucial for regulating cancer progression and cisplatin-resistance; however, all our current data were based on the preliminary cellular and animal experiments, future clinical analysis was still needed to verify those results and conclusions in clinic.

## Conclusions

Taken together, analysis of data in this study evidenced that miR-6734-3p served as a tumor suppresser to hinder NSCLC progression by degrading ZEB2 mRNA. Also, both miR-6734-3p overexpression and ZEB2 silence increased cisplatin-sensitivity in NSCLC cells.

## Supplementary Material

Supplemental Material
