# Supplementary material for: Targeting the miR-6734-3p/ZEB2 axis hampers development of non-small cell lung cancer (NSCLC) and increases susceptibility of cancer cells to cisplatin treatment
Source: Bioengineered. 2021 Jun 9;12(1):2499–510. doi: 10.1080/21655979.2021.1936891 (PMC8806905; doi:10.1080/21655979.2021.1936891)
Supplement: Supplemental Material [file KBIE_A_1936891_SM4803.zip › Supplementary Figures caption.docx]

**Supplementary Figures**

Figure S1. Overexpression of ZEB2 rescued cell proliferation in high-dose cisplatin treated (A) A549 cells and (B) H1299 cells. Individual experiment had 3 repetitions, and **P* < 0.05.

Figure S2. The wild-type and mutant ZEB2 were delivered into the NSCLC cells, and cell proliferation was evaluated by performing MTT assay. Individual experiment had 3 repetitions, and **P* < 0.05.

Figure S3. Real-Time qPCR was performed to measure miR-6734-3p levels in the serum collected from NSCLC patients and normal volunteers.
