# Supplementary figures and images for: Targeting the miR-6734-3p/ZEB2 axis hampers development of non-small cell lung cancer (NSCLC) and increases susceptibility of cancer cells to cisplatin treatment
Source: Bioengineered. 2021 Jun 9;12(1):2499–510. doi: 10.1080/21655979.2021.1936891 (PMC8806905; doi:10.1080/21655979.2021.1936891)

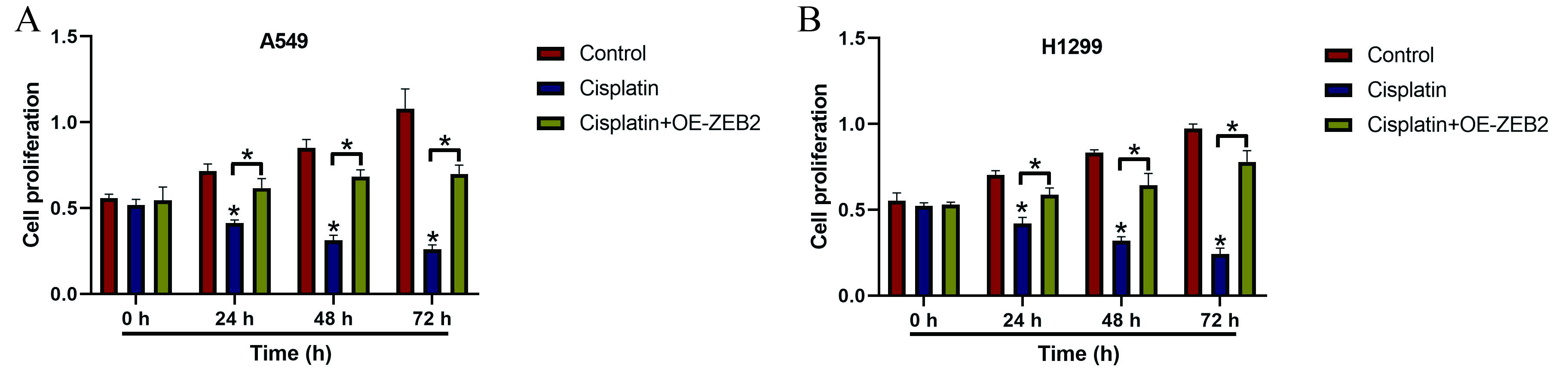

Supplement: Supplemental Material [file KBIE_A_1936891_SM4803.zip › Figure S1.jpg]

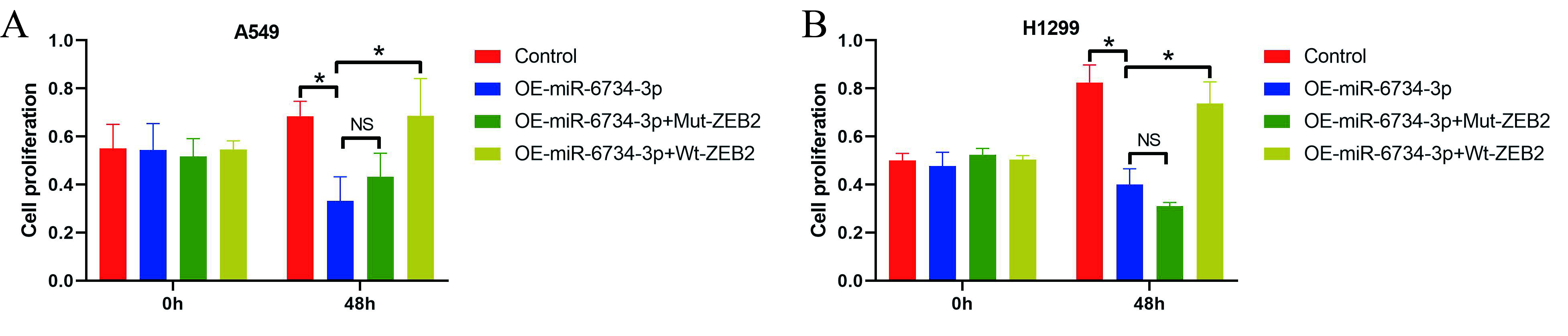

Supplement: Supplemental Material [file KBIE_A_1936891_SM4803.zip › Figure S2.jpg]

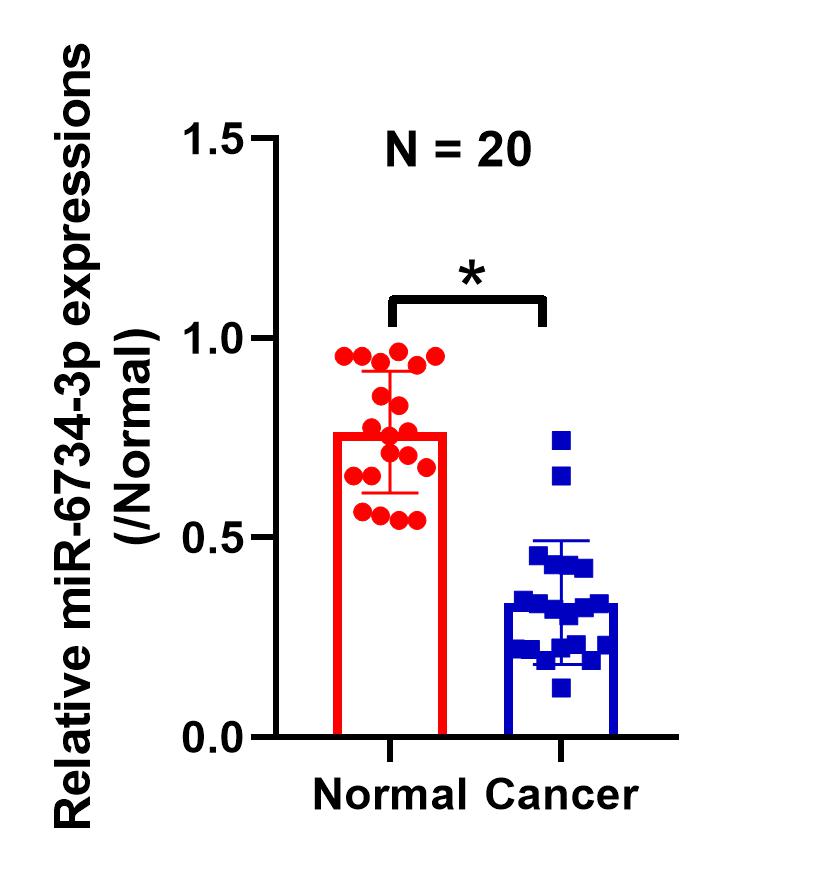

Supplement: Supplemental Material [file KBIE_A_1936891_SM4803.zip › Figure S3.jpg]
